# Lcn2 secreted by macrophages through NLRP3 signaling pathway induced severe pneumonia

**DOI:** 10.1093/procel/pwae045

**Published:** 2024-08-24

**Authors:** Mingya Liu, Feifei Qi, Jue Wang, Fengdi Li, Qi Lv, Ran Deng, Xujian Liang, Shasha Zhou, Pin Yu, Yanfeng Xu, Yaqing Zhang, Yiwei Yan, Ming Liu, Shuyue Li, Guocui Mou, Linlin Bao

**Affiliations:** Beijing Key Laboratory for Animal Models of Emerging and Reemerging Infectious Diseases, NHC Key Laboratory of Comparative Medicine, Institute of Laboratory Animal Science, CAMS & PUMC, Beijing 100021, China; Beijing Key Laboratory for Animal Models of Emerging and Reemerging Infectious Diseases, NHC Key Laboratory of Comparative Medicine, Institute of Laboratory Animal Science, CAMS & PUMC, Beijing 100021, China; National Center of Technology Innovation for Animal Model, Beijing 100021, China; Beijing Key Laboratory for Animal Models of Emerging and Reemerging Infectious Diseases, NHC Key Laboratory of Comparative Medicine, Institute of Laboratory Animal Science, CAMS & PUMC, Beijing 100021, China; National Center of Technology Innovation for Animal Model, Beijing 100021, China; Beijing Key Laboratory for Animal Models of Emerging and Reemerging Infectious Diseases, NHC Key Laboratory of Comparative Medicine, Institute of Laboratory Animal Science, CAMS & PUMC, Beijing 100021, China; National Center of Technology Innovation for Animal Model, Beijing 100021, China; Beijing Key Laboratory for Animal Models of Emerging and Reemerging Infectious Diseases, NHC Key Laboratory of Comparative Medicine, Institute of Laboratory Animal Science, CAMS & PUMC, Beijing 100021, China; National Center of Technology Innovation for Animal Model, Beijing 100021, China; Beijing Key Laboratory for Animal Models of Emerging and Reemerging Infectious Diseases, NHC Key Laboratory of Comparative Medicine, Institute of Laboratory Animal Science, CAMS & PUMC, Beijing 100021, China; National Center of Technology Innovation for Animal Model, Beijing 100021, China; Beijing Key Laboratory for Animal Models of Emerging and Reemerging Infectious Diseases, NHC Key Laboratory of Comparative Medicine, Institute of Laboratory Animal Science, CAMS & PUMC, Beijing 100021, China; National Center of Technology Innovation for Animal Model, Beijing 100021, China; Beijing Key Laboratory for Animal Models of Emerging and Reemerging Infectious Diseases, NHC Key Laboratory of Comparative Medicine, Institute of Laboratory Animal Science, CAMS & PUMC, Beijing 100021, China; Beijing Key Laboratory for Animal Models of Emerging and Reemerging Infectious Diseases, NHC Key Laboratory of Comparative Medicine, Institute of Laboratory Animal Science, CAMS & PUMC, Beijing 100021, China; National Center of Technology Innovation for Animal Model, Beijing 100021, China; Beijing Key Laboratory for Animal Models of Emerging and Reemerging Infectious Diseases, NHC Key Laboratory of Comparative Medicine, Institute of Laboratory Animal Science, CAMS & PUMC, Beijing 100021, China; National Center of Technology Innovation for Animal Model, Beijing 100021, China; Beijing Key Laboratory for Animal Models of Emerging and Reemerging Infectious Diseases, NHC Key Laboratory of Comparative Medicine, Institute of Laboratory Animal Science, CAMS & PUMC, Beijing 100021, China; Beijing Key Laboratory for Animal Models of Emerging and Reemerging Infectious Diseases, NHC Key Laboratory of Comparative Medicine, Institute of Laboratory Animal Science, CAMS & PUMC, Beijing 100021, China; Beijing Key Laboratory for Animal Models of Emerging and Reemerging Infectious Diseases, NHC Key Laboratory of Comparative Medicine, Institute of Laboratory Animal Science, CAMS & PUMC, Beijing 100021, China; Beijing Key Laboratory for Animal Models of Emerging and Reemerging Infectious Diseases, NHC Key Laboratory of Comparative Medicine, Institute of Laboratory Animal Science, CAMS & PUMC, Beijing 100021, China; Beijing Key Laboratory for Animal Models of Emerging and Reemerging Infectious Diseases, NHC Key Laboratory of Comparative Medicine, Institute of Laboratory Animal Science, CAMS & PUMC, Beijing 100021, China; Beijing Key Laboratory for Animal Models of Emerging and Reemerging Infectious Diseases, NHC Key Laboratory of Comparative Medicine, Institute of Laboratory Animal Science, CAMS & PUMC, Beijing 100021, China; National Center of Technology Innovation for Animal Model, Beijing 100021, China; State Key Laboratory of Respiratory Health and Multimorbidity, Beijing 100005, China

**Dear Editor,**

The case fatality rate of early SARS-CoV-2 infection is 3% (Ghebreyesus, 2020), and the severe case rate is 24.3% (Sun et al., 2020). From the prototypic SARS-CoV-2 strain to the emergence of Alpha, Beta, and Delta variants, which ultimately led to the outbreak of Omicron variants, these strains have undergone a series of evolutionary changes. Starting from BA.1, BA.2, BA.4, and BA.5 lineage to XBB lineage before branching out into the current dominant JN.1 lineage, this has resulted in a significant decrease in severe case rate from 24.3% to 0.3% (Horita and Fukumoto, 2023). Despite this downward trend in severe pneumonia rate caused by SARS-CoV-2 infection, there is still much that remains unclear about its underlying mechanisms.

While the wild-type mice are not susceptible to direct infection by the prototypic SARS-CoV-2 strain, hACE2 transgenic mice can be infected, resulting in pulmonary lesions characterized primarily by inflammatory cell infiltration, thickening of the alveolar septum, epithelial cell damage, and focal bleeding, ultimately leading to a manifestation of mild or moderate pneumonia (Bao et al., 2020; Sun et al., 2020). After infection with SARS-CoV-2, degranulation of lung mast cells is observed in hACE2 transgenic mice and rhesus monkeys, leading to the production of inflammatory cytokines including IL-6, IL-8, IL-1β, TNF-α, and infiltration of inflammatory cells, thereby triggering an inflammatory storm (Wu et al., 2021). In addition, upon SARS-CoV-2 infection, macrophages are found to be activated through MyD88 and TIRAP pathways causing accumulation of lung macrophages along with secretion of TNF-α, IL-lβ, IL-6, and MCP-1. All the aforementioned studies are predicated on the characteristics of lung lesions post-infection and the underlying mechanisms precipitating pulmonary injury (Lai et al., 2023). Nevertheless, managing severe pneumonia in patients with SARS-CoV-2 infection poses a formidable clinical challenge. Current animal models typically induce only moderate or mild pneumonia following infection (Bao et al., 2020; Sun et al., 2020), thus impeding accurate simulation of severe pneumonia manifestations and limiting exploration into its underlying mechanisms.

In this study, the Beta variant (P0) was adapted to BALB/c mice to generate a viral adaptation strain (P7) exhibiting enhanced virulence. Following infection with the adaptation strain, mice displayed exacerbated lung lesions characterized by severe interstitial pneumonia, notable alveolar septum widening accompanied by infiltration of inflammatory cells dominated by macrophages, and formation of hyaline membrane within the alveoli. Consequently, respiratory disorders ensued leading to rapid mortality in mice. Establishing this wild-type mouse model of severe pneumonia holds paramount significance for investigating the mechanisms underlying severe illness induced by viral infection caused by SARS-CoV-2, identifying biomarkers associated with severe illness, revealing pivotal targets or crucial time windows for intervention, and potentially optimizing or discovering novel therapeutic approaches.

P0 strain was passed in mice for seven consecutive generations, an adapted strain was obtained, which was designated as the P7 strain (Fig. S1A and S1B). Then, the mice were intranasally administered with a non-lethal dose of 50 µL (10^3.9^ median tissue culture infective dose, TCID_50_) of P0 or P7 virus strain, and symptoms were monitored daily (Fig. S1C). The body weight in the P0 group decreased by 2.65% at 3 days post-infection (dpi) before gradually recovering (Fig. S1D). The peak viral load in the lungs of the P0 group was observed at 3 dpi, reaching 10^7.51^ copies/mL (Fig. S1E), and the lungs exhibited moderate interstitial pneumonia at 3 and 5 dpi (Fig. S1F). Notably, the severity of lung lesions in the P7 group was most pronounced at 5 dpi, and a significant average weight loss rate of 20.24% was observed at 5 dpi (Fig. S1D). At 3 and 5 dpi, the viral replication reached its peak in the P7 group, with viral loads of 10^8.71^ TCID_50_/mL and 10^9.01^ TCID_50_/mL, respectively (Fig. S1E). After mice were infected with the P7 strain, the duration of viral replication was prolonged, and the lower respiratory tract exhibited an augmented capacity for viral replication. Moreover, the lung lesions were characterized by the presence of substantial exudates, consisting of inflammatory cells and edematous fluid in the alveoli, especially, the formation of hyaline membrane was observed (Fig. S1F), which persisted until 7 dpi without remission. Pathological scores revealed that the overall severity and duration of pulmonary interstitial pneumonia were greater in the P7 group compared to the P0 group (Fig. S1G). Furthermore, we sequenced the P7 strain and identified two mutations in the S segment compared to the P0 strain, specifically I68K and W682R (Fig. S1B). Notably, the W682R mutation is located proximal to the furin cleavage site at the S1/S2 junction. Following the invasion of host cells by the SARS-CoV-2 virus, furin protease facilitates cleavage at both the S1/S2 and S2' sites on the S protein, thereby promoting viral fusion with target cells. Previous studies have demonstrated that a mutation at position P681R near the furin cleavage site enhances the efficiency of furin protease in cleaving the S protein, consequently accelerating fusion with target cells and leading to increased pathogenicity (Saito et al., 2022). Based on this evidence, we hypothesize that mutation occurring at residue W682R may similarly enhance furin protease’s cutting efficiency and expedite viral fusion with target cells, ultimately facilitating viral invasion. The results obtained from our *in vivo* and *in vitro* experiments demonstrated that the P7 strain significantly augmented both virulence and replication capacity.

By detecting the mRNA levels of inflammatory cytokines in the lungs at 3, 5, and 7 dpi, it was observed that the expression levels of IL-6, TNF-α, CXCL1, CXCL2, MCP-1, and IL-1β were significantly elevated in the P7 group compared to the P0 group (Fig. S2A). In addition, analysis of cytokine levels in mouse serum and lung homogenates revealed a substantial increase in various cytokines and chemokines following infection with P7 strain, reaching their peak at 3 dpi. At 3 dpi, the levels of various cytokines, including TNF-α, IL-6, IL-1α, IFN-γ, as well as chemokines, including KC, MIG, MCP-1, MIP-1α, and MIP-1β were significantly elevated in the lungs of mice infected with P7 strain (Fig. S2B). Similarly, the trend of cytokine level changes in mouse serum was consistent with that observed in the lung (Fig. S2C). In conclusion, after infection in mice, the P7 strain induced a significant upregulation of chemokines and cytokines in both lung and serum, suggesting that P7 infection elicits a pronounced systemic inflammation beyond pulmonary involvement.

Then, we conducted RNA-sequencing (RNA-Seq) analysis on the lungs of mice infected with P0 and P7 strain, consistent with findings from the infection experiments, the principal component analysis (PCA) results, based on the differentially expressed genes (DEG), indicated that the P0 group did not form a distinct cluster separate from the control group (Fig. S3A). In contrast, mice infected with the P7 strain formed a distinct cluster on the first principal component (eigenvalue 1 = 0.2563). Furthermore, the second principal component of PCA (eigenvalue 2 = 0.1076) further divided P7 virus-infected mice into two subgroups: one at 3 dpi and the other at 5 and 7 dpi (Fig. S3A). Based on the transcriptomic data analysis, 2,919 (1,814 upregulated, 1,095 downregulated) DEG had been identified in the 5 dpi, respectively (*P* value < 0.05, |log_2_(Fold Change)| > log_2_(1.5)) (Fig. S3B). We focused specifically on the transcriptional outcomes of P0 and P7 strain-infected mice at 5 dpi, aiming to precisely identify evidence indicating that the P7 strain led to more severe symptoms in mice. It is worth noting that the *Lcn2* gene exhibited significant differences at 3, 5, and 7 dpi (Fig. 1A). In the realm of viral infections, Lcn2 exhibits a close correlation with disease severity in patients afflicted with influenza (Huang et al., 2022), displaying markedly elevated levels in severe cases compared to mild or moderate ones. The analysis of immune-related protein expression in the serum of COVID-19 patients reveals a significant upregulation in Lcn2 level compared to healthy individuals (Abers et al., 2021). Due to its biological roles in a variety of diseases, we have identified the Lcn2 gene as a candidate gene of particular interest for further investigation.

**Figure 1.**
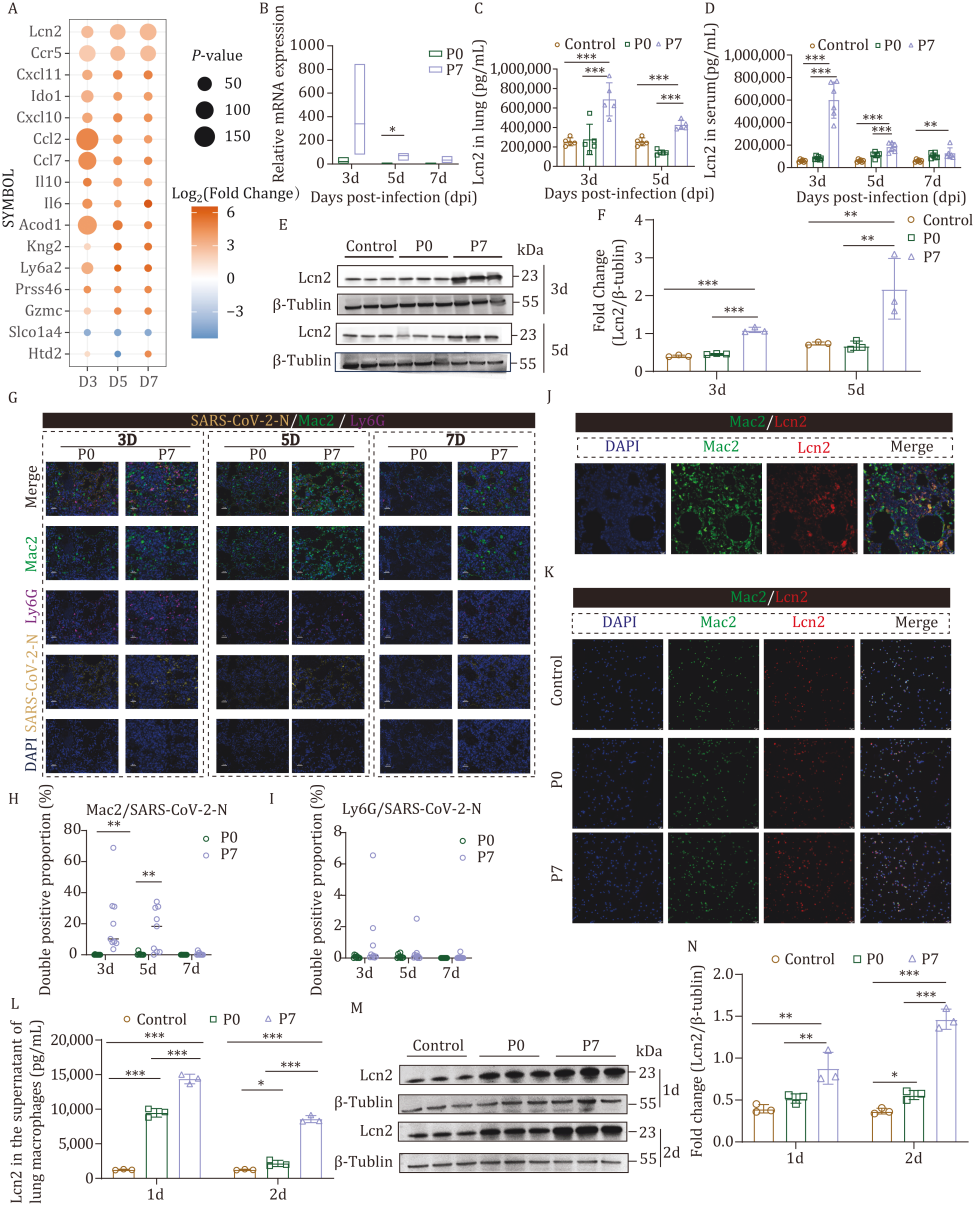
P7 stain infection significantly augmented the secretion of Lcn2 in macrophages. After infecting BALB/c mice with the P0 strain and P7 strain, the lung was collected at 3, 5, and 7 dpi for RNA-Seq analysis, Western blot, ELISA, and multiplex IHC staining. (A) The differential expression of genes at various time points following infection with the P7 mutant strain compared to the P0 strain. The size of the circles represents −log_10_(*P* value), while the color indicates log_2_(Fold Change). (B) Lcn2 mRNA expression in the lungs of infected mice was detected by qRT-PCR at 3, 5, and 7 dpi (*n* = 3). (C) Lcn2 protein expression in lung homogenates of infected mice was detected by ELISA at 3 and 5 dpi (*n* = 5). (D) Lcn2 protein expression in the serum of infected mice was detected by ELISA at 3, 5, and 7 dpi (*n *= 6). (E and F) Lcn2 protein expression in the lungs of infected mice was detected by Western blot at 3 and 5 dpi (*n = *3). (G) Co-localization of the SARS-CoV-2 viral antigen (N protein, the fourth line) with macrophages (Mac2, the second line) and neutrophils (Ly6G, the third line) in the lungs of infected mice was assessed by multiplex IHC staining. (H) The ratio of Mac2 to SARS-CoV-2-N double-positive cells was determined (*n = *9). (I) The ratio of Ly6G to SARS-CoV-2-N double-positive cells was determined (*n = *9). (J) The co-localization results of lung macrophages (Mac2, the second pannel) and Lcn2 (the third pannel) were analyzed by immunofluorescence. (K) The co-localization results of sorted macrophages (Mac2, the second pannel) and Lcn2 (the third pannel) from the lungs of mice in each group (control, P0, and P7 group) were analyzed by cell immunofluorescence. (L) Lcn2 protein expression in the supernatant of macrophages sorted from the lungs of mice in each group (control, P0, and P7 group) was detected by ELISA at 1 dpi and 2 dpi (*n = *3). (M and N) Lcn2 protein expression in the lungs of mice in each group (control, P0, and P7 group) was detected by Western blot at 1 and 2 dpi (*n* = 3). Bar = 25 μm or 10 μm. Significant differences are indicated with asterisks (*P < 0.05; **P < 0.01; ***P < 0.001; Student’s *t*-test or one-way ANOVA analysis).

The mRNA expression level of the Lcn2 in the lungs was assessed at 3, 5, and 7 dpi in group P0 and P7. Notably, it was observed that compared to the P0 group, the mRNA level of the Lcn2 exhibited upregulation at various time points in mice infected with P7 strain (Fig. 1B). Besides, the level of Lcn2 in the serum of P7 group was significantly higher than that in the P0 group at 3 dpi and 5 dpi (*P* < 0.001) (Fig. 1D). Hence, there was a robust positive correlation between the Lcn2 protein expression level in serum and the progression of disease in mice during viral infection. The level of Lcn2 protein in the lungs of mice infected with P0 or P7 strain at 3 and 5 dpi were quantified using enzyme-linked immunosorbent assay (ELISA) and Western blot, respectively. ELISA results revealed a significant increase in Lcn2 protein level in lung homogenates after infection with P7 compared to the P0 group at 3 and 5 dpi (*P* < 0.001) (Fig. 1C). Similar results were obtained by Western blot analysis (Fig. 1E and 1F). Moreover, mRNA and protein expression levels of Lcn2 were consistently higher in the P7-infected group compared to the P0 group across different time points, which was consistent with our transcriptome data.

Next, multiplex IHC staining was performed on the lung pathological sections of mice at different time points post-infection. The results demonstrated an increased number of macrophages and neutrophils in the lungs of the P7 group compared to the P0 group at 3 dpi following infection. Notably, at 5 dpi, there was a significant increase in macrophage infiltration observed specifically in the P7 group (Fig. 1G). We conducted statistical analysis on SARS-CoV-2-N and Mac2 double-positive cells, as well as SARS-CoV-2-N and Ly6G double-positive cells at various time points post-infection. Our findings revealed a significant augmentation in the population of SARS-CoV-2-N and Mac2 double-positive cells within the P7 group compared to the P0 group at 3 dpi and 5 dpi (*P* < 0.01) (Fig. 1H). At 3, 5, and 7 dpi, the proportion of SARS-CoV-2-N and Ly6G double-positive cells in the P7 group was slightly higher than that in the P0 group (Fig. 1I). This observation suggests an increase in macrophages infiltration in P7 group, potentially indicating its association with pneumonia severity.

Studies have demonstrated that in patients with atherosclerosis, Lcn2 can also be synthesized by TNF-α-stimulated macrophages, thereby enhancing the mRNA expression of M1 macrophage markers including TNF-α, iNOS, IL-6, and CCL5 (Oberoi et al., 2015). Interestingly, single-cell sequencing analysis of lungs and whole blood samples obtained from influenza virus-infected patients reveal that Lcn2 is predominantly generated by neutrophils while being expressed in other myeloid cells as well (Huang et al., 2022). To determine the source of Lcn2 in the lung following P7 infection, we conducted immunofluorescence staining. The results demonstrated co-localization of Lcn2 with the macrophage marker Mac2 (Fig. 1J), suggesting that macrophages may be responsible for producing Lcn2. Subsequently, macrophages were isolated from the lungs of mice infected with P0 and P7 virus strain at 2 dpi, and targeted immunofluorescence staining was performed on macrophages. Interestingly, a higher proportion of macrophages in the P7 group exhibited expression of Lcn2 (Fig. 1K). The selected macrophages were cultured *in vitro* to detect the expression level of Lcn2 protein in both the supernatant and cells. Compared to the control group and P0 group, there was a significant increase in Lcn2 expression level (Fig. 1L). Western blot analysis showed a comparable expression level of Lcn2 in macrophages. Significantly higher protein levels of Lcn2 were observed in the P7 group compared to both the control and P0 groups (Fig. 1M and 1N). Our findings suggest that macrophages serve as the primary source of Lcn2 production in the lungs of mice infected with the P7 strain.

Previous studies have revealed that the expression of Lcn2 is upregulated in SWA-infected macrophages, and Western blot analysis reveals activation of the NF-κB signaling pathway characterized by increased phosphorylation of NF-κB and significant degradation of IκBα. Furthermore, treatment with BAY11-7082 results in the downregulation of Lcn2 protein expression (Shen et al., 2021). To further explore the mechanism by which macrophages produce Lcn2 in mice infected with the virus, we initially performed bioinformatics analysis. It was evident that the expression levels of some of the genes were significantly elevated in the P7-treated samples (Fig. 2A). These genes sets can aid us in the initial screening of DEGs. The gene ontology (GO) enrichment results for DEGs showed that infection with the P7 strain induced strong transcription of genes related to antiviral response, inflammation, cytokine production, and cell adhesion in mice. We particularly focused on ontology terms related to biological processes. By identifying common pathways across three-time points, we discovered significant enrichment terms associated with *Lcn2* gene, which were associated with response to virus, defense response to bacterium and cellular response to chemical stress respectively (Fig. S3D). The common DEG we identified in these pathways were *Nlrp3*, *Caspase1*, and *Pycard*, which was consistent with the results of gene expression levels. Besides, according to our experimental objectives, we focused on the Kyoto Encyclopedia of Genes and Genomes (KEGG) subcategory related to the “Immune system” of Organismal Systems, “Infectious disease: viral” and “Infectious disease: bacterial” of Human Diseases. These enrichment terms revealed that infection with the P7 mutant strain induced strong transcription of genes related to antiviral response, cytokine receptor interaction, and Neutrophil Extracellular Trap formation in mice (Fig. S3E). The *Lcn2* gene was found to be associated with the IL-17 signaling pathway based on the KEGG enrichment analysis, while its expression was found to be probably regulated by the NF-κB pathway. In this pathway, the upregulation of *Nf-*κ*b* and *Ap-1* gene expression led to the upregulation of *Il-1β* precursor, thereby generating more mature *Il-1β* genes, and induced the upregulation of genes such as *Il-6*, *Tnf-α*, *Lcn2*, *Cxcl* gene family, and *Ccl* gene family. However, except for *pro-Il-1β* and *Il-1β*, no statistically significant differences were observed in the expression of other genes within the NF-κB pathway (Fig. 2B). Based on this, we postulate that the upregulation of *Lcn2* expression might be correlated with *Il-1β*. Therefore, we further investigated all pathways that included *Il-1β* and were related to inflammatory responses. The statistical results showed that the NOD-like receptor signaling pathway was enriched not only by the differential genes between mice infected with the P0 and P7 strain but also had the smallest average *P* value of the included differential genes (Fig. S3C). Hence, we believe that this pathway is most closely related to the elevated *Il-1β* gene, which leads to a specific increase in *Lcn2* gene expression. Network analysis of genes involved in this pathway demonstrated a focus on the pro-inflammatory effects mediated by the *Nlrp3* regulatory axis, in which the related genes were significantly upregulated (Fig. 2B). The upregulation of the *Nlrp3* gene results in overexpression of *Caspase1*, thereby accelerating the efficiency of the converting *Il-1β* precursor to mature *Il-1β* gene. Given the upregulation of *Nlrp3*, *Asc*, and *pro-Caspase1* in the *Nlrp3* regulatory axis, we postulate that *Lcn2* expression may be governed by the NOD-like pathway’s *Nlrp3* regulatory axis. In addition, based on transcriptomic findings, a strong potential correlation between *Nlrp3* and *Lcn2* expression was observed, the transcripts per million (TPM) of P7 group was significantly higher than that of P0 group and control group (Fig. 2C and 2D), thereby potentially influencing lung infection in mice. Therefore, to further validate its association with Lcn2 production, we focused on investigating the NLRP3 signaling pathway based on the enrichment results from GO and KEGG analyses.

**Figure 2.**
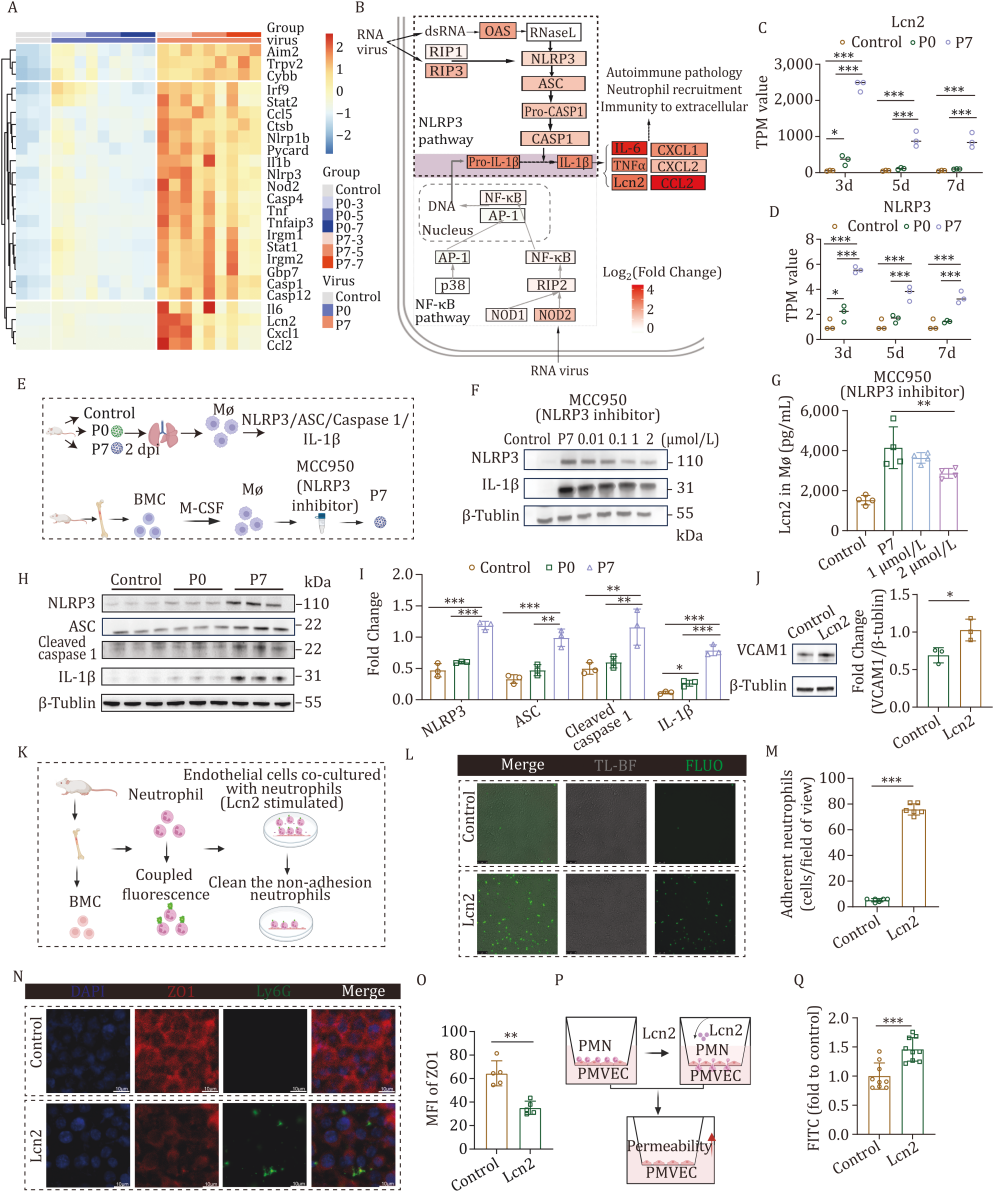
Lcn2 was produced by macrophages through the NLRP3 signaling pathway and led to compromised endothelial integrity. (A) Heatmap generated using TPM values of genes in the *Nlrp3*-*Lcn2* regulatory axis. The TPM values of all genes were normalized to a common standard using *Z*-score transformation. (B) Diagram illustrating the immune response pathways triggered by RNA viruses through the NLRP3 or NF-κB pathways. The lower dashed box represents the NF-κB pathway, the upper dashed box represents the NLRP3 pathway, and the shaded area represents the common parts of both pathways. The color of the genes indicates the log_2_(Fold Change) value of gene expression after infection in mice by P0 and P7 strain. (C) Expression of *Lcn2* in subgroups (TPM). (D) Expression of *Nlrp3* in subgroups (TPM). (E) Experimental design and sample collection. The upper panel: diagram illustrating the detection of proteins involved in the NLRP3 signaling pathway. The lower panel: schematic diagram of marrow macrophages infected with P7 strain treated with NLRP3 inhibitors (Mø, Macrophage; BMC, bone marrow cell). (F) The expression of NLRP3 and IL-1β was suppressed upon treatment of mouse bone marrow-derived macrophages with varying concentrations of MCC950, in addition to P7 strain infection at 0.0001 MOI. (G) After treating mice bone marrow-derived macrophages with MCC950 at concentrations of 1 and 2 μmol/L, followed by infection with P7 strain at 0.0001 MOI, the expression level of Lcn2 was quantified using ELISA (*n = *4). (H and I) The NLRP3, ASC, Cleaved caspase1, and IL-1β protein expression levels in sorted lung macrophages from each group of mice (control, P0, and P7 group) were detected through Western blot (*n = *3). The ordinate values were the ratio of NLRP3, ASC, Cleaved caspase1, and IL-1β to β-tubulin, respectively. (J) Expression of VCAM1 protein in PMVEC stimulated by Lcn2. (K) Schematic diagram of neutrophil adhesion experiment. (L) Neutrophils labeled with FITC fluorescence were co-cultured with endothelial cells, followed by a 2-hour stimulation with Lcn2. After removing the non-adherent neutrophils, the adhesion of neutrophils was observed under a fluorescence microscope. (M) Neutrophils labeled with FITC fluorescence were co-cultured with endothelial cells, followed by a 2-h stimulation with Lcn2. After removing the non-adherent neutrophils, the number of neutrophils adhering to endothelial cells within the visual field was quantified (*n = *6). (N) Following co-culture of neutrophils and endothelial cells, stimulation with Lcn2 was performed and the expression of ZO1 was analyzed via immunofluorescence (ZO1, the second pannel; Ly6G, the third pannel). (O) Mean fluorescence intensity (MFI) of ZO1 (*n = *5). (P) Transwell schematic representation. (Q) After the establishment of confluent monolayers of mouse lung microvascular endothelial cells, neutrophils were co-cultured and stimulated with Lcn2 for 24 h. Subsequently, FITC-Dextran was introduced, and the fluorescence intensity indicating leakage into the lower chamber was quantified using fluorescent enzyme labeling (*n = *9). Black bar = 75 μm and white bar = 10 μm. Significant differences are indicated with asterisks (*P < 0.05; **P < 0.01; ***P < 0.001; Student’s *t*-test or one-way ANOVA analysis).

Subsequently, the mRNA levels of NLRP3 and other proteins associated with signaling pathways in macrophages from the lung were detected. The results demonstrated a significant increase in the mRNA levels of NLRP3, ASC, Caspase1, and IL-1β in the P7 group compared to both the P0 and control group (*P* < 0.01) (Fig. S4A). Western blot analysis further confirmed these findings by revealing significantly elevated protein expression levels of NLRP3, ASC, Cleaved caspase1, and IL-1β in the P7 group when compared to the P0 group (*P* < 0.001 for NLRP3; *P *< 0.01 for Cleaved caspase1 and ASC; *P < *0.001 for IL-1β) (Fig. 2E, 2H, and 2I). In order to further clarify the mechanism of Lcn2 production, macrophages were pretreated with 2 μmol/L MCC950 before infection with the P7 strain (Fig. 2E). Subsequent analysis revealed significant inhibition of NLRP3 and IL-1β expression upon application of the NLRP3 inhibitor (Fig. 2F). Moreover, there was a notable reduction in the release of Lcn2 by macrophages into the supernatant (*P* < 0.01) (Fig. 2G). These findings strongly suggest that macrophages infected with the P7 strain may secrete Lcn2 via activation of the NLRP3 signaling pathway.

To investigate the impact of Lcn2 on the virulence of P7 strain, we employed Lcn2 to stimulate pulmonary microvascular endothelial cells (PMVEC) and observed a significant increase in mRNA expression levels of IL-6 (*P < *0.01), MIP-1α (*P < *0.05), IL-1α (*P < *0.01), and CXCL1 (*P < *0.05) after 24 h of stimulation with Lcn2 (Fig. S4B). These findings indicate that Lcn2 can potentiate inflammation by activating endothelial cells. In addition, Lcn2 stimulation significantly upregulated the mRNA expression levels of ICAM1 and VCAM1 in endothelial cells (Fig. S4C), while also enhancing the protein expression of VCAM1 (Fig. 2J). As crucial intercellular adhesion molecules, ICAM1 and VCAM1 play a pivotal role in facilitating firm leukocyte adhesion and transendothelial migration (Reglero-Real et al., 2016). After infection of PMVEC with HP-PRRSV, the expression of ICAM1 and VCAM1 is upregulated, thereby enhancing monocyte adhesion and rolling along the vascular wall, as well as compromising endothelial cell permeability (Sun et al., 2022). Subsequently, we co-cultured neutrophils with endothelial cells and observed that Lcn2 stimulation markedly augmented the adhesion between neutrophils and endothelial cells compared to the control group (*P* < 0.001) (Fig. 2K–M). Furthermore, following Lcn2 stimulation of endothelial cells, the expression of intercellular connections and the integrity of intercellular connections were diminished (Fig. 2N and 2O). Utilizing FITC-Dextran as a tracer, the fluorescence intensity of leaked FITC into the lower chamber of transwell after Lcn2 stimulation was significantly elevated compared to that in the control group (*P < *0.001) (Fig. 2P and 2Q), suggesting that Lcn2 stimulation disrupts tight junctions between endothelial cells and enhances endothelial cell permeability.

Taken together, this study revealed a significant upregulation of Lcn2 in P7-infected mice, with its expression pattern closely mirroring disease progression. By identifying Lcn2 as a pivotal protein warranting further investigation, we discovered its ability to induce the secretion of inflammatory mediators by endothelial cells and enhance neutrophil-endothelial cell adhesion, thereby exacerbating pulmonary inflammation in P7-infected mice and augmenting the pathogenicity of the P7 strain. Finally, Lcn2 exhibited a strong correlation with severe SARS-CoV-2 infection, necessitating further investigation to explore the precise role of Lcn2 in SARS-CoV-2 infection. Consequently, it has the potential to emerge as a novel target for both diagnosis and treatment strategies against severe COVID-19.

## Supplementary data

Supplementary data is available at https://doi.org/10.1093/procel/pwae045.

pwae045_suppl_Supplementary_Material

## Footnotes

We thank Professor Weifeng Shi from Shanghai Jiao Tong University for assistance in sequence identification and the Beijing Center for Disease Prevention and Control Virus Disease Prevention and Control Institute for providing GDPCC nCoV84 as a gift. All animal procedures were carried out in accordance with institutional guidelines and approved by the Institutional Animal Care and Use Committee (IACUC) of the Institute of Laboratory Animal Science (ILAS) and Peking Union Medical College (PUMC) (BLL20013 and BLL23011). All the authors declare their agreement to participate. All the authors give their consent for the publication of all materials within the text in *Protein & Cell*.

Conceptualization: L.L.B.; Methodology: M.Y.L., F.F.Q., and J.W., Investigation: M.Y.L., F.F.Q., J.W., F.D.L., Q.L., R.D., X.J.L., S.S.Z., P.Y., Y.F.Y., Y.Q.Z., Y.W.Y., M.L., S.Y.L., G.C.M., and C. Q., Writing: M.Y.L., F.F.Q., and L.L.B.; Funding Acquisition: L.L.B. and F. F. Q.; Supervision: L. L. B.

This work was supported by the National Research and Development Project of China (grant no. 2023YFF0724800), the CAMS Initiative for Innovative Medicine of China (grant no. 2021-I2M-1-035), the Sector Fund (2060302), and Young Elite Scientists Sponsorship Program by CAST (YESS) (grant no: 2020QNRC001).

All raw data are available from the corresponding author upon reasonable request. Source data are provided with this paper as a source data file.

## References

[CIT0001] Abers MS , Delmonte OM, Ricotta EE et al NIAID COVID-19 Consortium. An immune-based biomarker signature is associated with mortality in COVID-19 patients. JCI Insight 2021;6:e144455. 33232303 10.1172/jci.insight.144455 PMC7821609

[CIT0002] Bao L , Deng W, Huang B et al The pathogenicity of SARS-CoV-2 in hACE2 transgenic mice. Nature 2020;583:830–833. 32380511 10.1038/s41586-020-2312-y

[CIT0003] Horita N , Fukumoto T. Global case fatality rate from COVID-19 has decreased by 96.8% during 2.5 years of the pandemic. J Med Virol 2023;95:e28231. 36253938 10.1002/jmv.28231 PMC9874414

[CIT0004] Huang Z , Li H, Liu S et al Identification of neutrophil-related factor Lcn2 for predicting severity of patients with influenza a virus and SARS-CoV-2 infection. Front Microbiol 2022;13:854172. 35495713 10.3389/fmicb.2022.854172 PMC9039618

[CIT0005] Lai D , Zhu K, Li S et al SARS-CoV-2 N protein triggers acute lung injury via modulating macrophage activation and infiltration in *in vitro* and *in vivo*. J Inflamm Res 2023;16:1867–1877. 37143821 10.2147/JIR.S405722 PMC10153437

[CIT0006] Oberoi R , Bogalle EP, Matthes LA et al Lipocalin 2 mediates pro-atherosclerotic processes and is elevated in patients with coronary artery disease. PLoS One 2015;10:e0137924. 26367277 10.1371/journal.pone.0137924 PMC4569430

[CIT0007] Reglero-Real N , Colom B, Bodkin JV et al Endothelial cell junctional adhesion molecules: role and regulation of expression in inflammation. Arterioscler Thromb Vasc Biol 2016;36:2048–2057. 27515379 10.1161/ATVBAHA.116.307610 PMC5035539

[CIT0008] Saito A , Irie T, Suzuki R et al Genotype to Phenotype Japan (G2P-Japan) Consortium. Enhanced fusogenicity and pathogenicity of SARS-CoV-2 Delta P681R mutation. Nature 2022;602:300–306. 34823256 10.1038/s41586-021-04266-9 PMC8828475

[CIT0009] Shen H , Wang Z, Huang A et al Lipocalin 2 is a regulator during macrophage polarization induced by soluble worm antigens. Front Cell Infect Microbiol 2021;11:747135. 34616693 10.3389/fcimb.2021.747135 PMC8489661

[CIT0010] Sun SH , Chen Q, Gu HJ et al A mouse model of SARS-CoV-2 infection and pathogenesis. Cell Host Microbe 2020;28:124–133.e4. 32485164 10.1016/j.chom.2020.05.020 PMC7250783

[CIT0011] Sun W , Wu W, Jiang N et al Highly pathogenic PRRSV-infected alveolar macrophages impair the function of pulmonary microvascular endothelial cells. Viruses 2022;14:452. 35336858 10.3390/v14030452 PMC8948932

[CIT0012] Wu ML , Liu FL, Sun J et al SARS-CoV-2-triggered mast cell rapid degranulation induces alveolar epithelial inflammation and lung injury. Signal Transduct Target Ther 2021;6:428. 34921131 10.1038/s41392-021-00849-0 PMC8677926

