# Supplementary material for: Lcn2 secreted by macrophages through NLRP3 signaling pathway induced severe pneumonia
Source: Protein Cell. 2024 Aug 24;16(2):148–55. doi: 10.1093/procel/pwae045 (PMC11786722; doi:10.1093/procel/pwae045)
Supplement: pwae045_suppl_Supplementary_Material [file pwae045_suppl_supplementary_material.zip › PAC-24196-BLL-Supplemental files.pdf]

Supplementary Materials for

Lcn2 secreted by macrophages through NLRP3 signaling pathway induced severe pneumonia

Mingya Liu[1†], Feifei Qi[1,2†], Jue Wang[1,2†], Fengdi Li[1,2], Qi Lv[1,2], Ran Deng[1,2], Xujian Liang[1,2], Shasha Zhou[1], Pin Yu[1,2], Yanfeng Xu[1,2], Yaqing Zhang[1], Yiwei Yan[1], Ming Liu[1], Shuyue Li[1], Guocui Mou[1], Linlin Bao[1,2,3*].

Correspondence to: bllmsl@aliyun.com

These authors contributed equally: Mingya Liu, Feifei Qi, Jue Wang

This PDF file includes:

Materials and Methods
Figures. S1 to S4
Table S1

MATERIALS AND METHODS

Ethics statement

Mouse studies were performed in an animal biosafety level 3 facility with high-efficiency particulate air (HEPA)-filtered isolators. All procedures in this experiment involving animals were reviewed and authorized by the Institutional Animal Care and Use Committee of the Institute of Laboratory Animal Science (ILAS), Peking Union Medical College (PUMC) (BLL20013 and BLL23011). All of the experiments complied with all relevant ethical regulations.

Virus and cells

The SARS-CoV-2 strain 501Y.V2-GDPCC nCoV84, CSTR:16698.06. NPRC 2.062100001. Seed SARS-CoV-2 stocks and virus isolation studies were performed in Vero E6 cells. Titers for SARS-CoV-2 were determined using a median tissue culture infectious dose ($TCID_{50}$) assay. The Mouse lung microvascular endothelial cells were purchased from Shanghai Zhong Qiao Xin Zhou Biotechnology Co., Ltd., which are maintained in Mouse lung microvascular endothelial cells complete culture medium(Zhong Qiao Xin Zhou, PCM-M-01, Shanghai). The PMVEC were purchased from Shanghai Qingqi Biotechnology Co., Ltd., which are maintained in Dulbecco's modified Eagle's medium (DMEM) (HyClone) supplemented with 10% fetal bovine serum (FBS), 50 IU/ml penicillin, and 50 µg/ml streptomycin, and cells are incubated at 37 °C, 5% CO_2.

Mouse adaptation of SARS-CoV-2 virus

Viral adaptation was achieved by lung-to-lung passage. Briefly, nine-month-old female BALB/c mice (n=2) were inoculated via the intranasal route with SARS-CoV-2 stock virus, at a dose of 10^5 $TCID_{50}$, after intraperitoneal administration of anesthesia with 2.5% tribromoethanol at 0.02 mL/g. At 3 dpi, the inoculated mice were euthanized to obtain lungs. Supernatants were then collected by centrifugation, labeled as passage 0 (P0) and sequentially thereafter as P1,….Pn, the mutant we pass on to the 7th generation labeled as P7. New mice were inoculated with 50µl lung homogenate, and the procedure was repeated (lung-to-lung passage). The infected animals were continuously observed daily to record body weights, clinical symptoms, and death.

Pathogenicity of virus in BALB/c mice

To compare the pathogenicity of wild-type with mouse-adapted viruses in vivo, female 6-8-week-old BALB/c mice (n=14 per group) were anesthetized and inoculated intranasally with

50μl ($10^{3.9}$ $TCID_{50}$) of each type of virus, respectively. Five mice were selected randomly from each group to monitor the signs of disease, weight loss, and mortality daily up to 14 dpi. The remaining nine mice in each group were euthanized at 3,5, and 7dpi., and blood samples and tissues were collected for subsequent analysis of viral replication, lung histology, and pro-inflammatory cytokines.

Preparation of homogenate supernatant

Tissue homogenates were prepared by homogenizing perfused tissues using an electric homogenizer for 2 min in DMEM. The homogenates were centrifuged at 8,000 rpm for 10 min at 4 °C. The supernatant was collected for viral load detection.

RNA extraction and qRT-PCR

Total RNA was extracted from tissue homogenates of organs using the RNeasy Mini Kit (Qiagen), and reverse transcription was performed using the PrimerScript RT Reagent Kit (TaKaRa) following the manufacturers' instructions. qRT–PCR reactions were performed using the PowerUp SYBG Green Master Mix Kit (Applied Biosystems), in which samples were processed in duplicate using the following cycling protocol: 50°C for 2 min, 95 °C for 2 min, followed by 40 cycles at 95 °C for 15 s and 60 °C for 30 s, and then 95 °C for 15 s, 60 °C for 1 min, 95 °C for 45 s. Primer sequences used for qRT-PCR analysis are listed in Table S1.

Chemokine & Cytokine analysis

LEGENDplexTM Proinflammatory Chemokine Panel (13-plex) (Mouse) and Inflammation Panel (13-plex) (Mouse) were utilized to analyze chemokines and cytokines in serum and lung samples from 6-8-week-old BALB/c according to manufacturer's protocol. 25μl of clarified lung samples or 25μl of 1:2 diluted serum samples were incubated with magnetic capture beads, washed, and incubated with detection antibodies and SA-PE. Cytokines were recorded on Beckman Cytoflexs and quantitated via comparison to a standard curve. LEGENDplexTM Data Analysis software was used for data collection and analysis.

Transcriptomic analysis

Expected counts were compiled from RSEM quantifications and imported into R for downstream analyses. In downstream analyses, genes were required to have a mean TPM (Transcripts Per Million) value greater than 1 in at least one sub-group. After removing low-expressed genes, a total of 40838 genes were obtained for subsequent analysis.

Identifying differentially expressed genes (DEGs) was performed using the R package DESeq2(Love et al., 2014). Covariate adjustment for virus type was carried out in a generalized linear model using the Wald test (p-value<0.05, $|\log_2(\text{Fold Change, FC})| > \log_2(1.5)$). After correcting for library size, normalized expression matrices were obtained for downstream analysis.

To gain a further understanding of the biological functions of DEGs, we utilized the R package cluster profile(Yu et al., 2012) to perform enrichment analysis based on the hypergeometric testing model for Gene Ontology (GO) terms and KEGG pathways. GSEA analysis was executed by the gsePathway function in the ReactomePA(Yu and He, 2016) package with 1000 maximal size of each gene set for analysis.

ELISA

Lcn2 levels were measured in blood, tissue homogenate, and cell supernatant by enzyme-linked immunosorbent assay (ELISA). Mouse Lipocalin-2/NGAL ELISA Kit(Boster, EK0854) was used, according to the manufacturer's instructions. Briefly, the sample was added to the enzyme-labeled plate, then incubated at 37℃ for 90 minutes. Subsequently, it was transferred to monoantibodies diluent and incubated again at 37℃ for 60 minutes. After being washed three times, Avidin-Biotin-Peroxidase Complex(1×) was introduced. 30 minutes later, TMB was added and incubated for 15 minutes, then added the termination solution immediately. And the absorbance was measured at 450 nm with a Microplate Reader (BMG LABTACH PHERAstar FSX).

Western Blotting

The total protein was extracted from cells and lung tissue using RIPA lysis buffer containing protease inhibitors (1×) and phosphatase inhibitors (1×). Samples were centrifuged at 13000 rpm for 30 min at 4℃, and the supernatant was collected immediately. 20 μg of total protein per sample was separated by 4%-12% ExpressPlus™ PAGE Gel (GenScript), then transferred gel to the PVDF membrane. After blocking with 5% Bovine Serum Albumin in PBST 1 h at room temperature, the blots were respectively incubated with anti-Lcn2 (Abcam, ab216462, 1:1000), anti-IL1β (Abcam, ab283818, 1:1000), anti-ASC (Cell Signaling Technology, 67824 1:1000), anti-Cleaved caspase1 (Cell Signaling Technology, 89332, 1:1000), anti-NLRP3 (Cell Signaling Technology, 15101, 1:1000), anti-β tublin (Cell Signaling Technology, 86298S, 1:1000) antibodies at 4 °C overnight. After washing membranes with TBST wash buffer, incubated with

Mouse IgG secondary antibody (Genetex, GTX213111-01, 1:15000) and Rabbit IgG secondary antibody (Genetex, GTX213110-01, 1:15000) for 1 h at room temperature. After five washes with TBST, electrogenerated chemiluminescence (ECL) (NCM Biotech, Suzhou) was employed to develop and fix the samples, followed by imaging using Tanon 5500 Chemiluminescent Imaging System (Tanon, Shanghai). The experiment was carried out three times.

Immunofluorescence

Paraffin sections (3-4μm in thickness) were dewaxed to water, and then antigen repair was performed. After five washes with PBS, 0.05%Tritox-100 penetrated for 10 min. Repeat with PBS for 5 washes. After blocking with goat serum in PBS 1 h at room temperature, the sections were respectively incubated with anti-Lcn2 (Abcam, ab216462, 1:2000), anti-Mac2 (CEDARLANE, CL8942AP, 1:600) antibodies at 4 °C overnight. The sections were rewarmed for 45 minutes at room temperature on the second day, after five washes with PBS, added the secondary antibody separately for 1 h at room temperature. Finally, DAPI seals the slices. The cell immunofluorescence starts with 0.05%Tritox-100, which is incubated with anti-ZO1 (Abcam, ab276131, 1:500) and anti-Lcn2. Images were finally captured using fluorescence microscopy(Leica).

Multiplex IHC staining

Multiplex IHC staining was performed on formalin-fixed, paraffin-embedded slides using an Opal multiplex IHC system (NEL811001KT, PerkinElmer) according to the manufacturer's instructions. Briefly, after slide preparation and heat-induced epitope retrieval, slides were blocked with PerkinElmer Antibody Diluent Block buffer. The following primary antibodies were used: anti-SARS-CoV-2-N (SinoBiological, 40143, 1:800), anti-Mac2 (CEDARLANE, CL8942AP, 1:600), anti-Ly6G (abcam, ab238132, 1:1000). Each slide was baked in the oven at 75°C for 1h. Then, the slides were deparaffinized with xylene and rehydrated through a graded series of ethanol solutions. After antigen retrieval in a microwave, the slides were washed in TBST wash buffer. After blocking, the sections were incubated with primary antibodies for 1h and then incubated with polymer HRP Ms+Rb as the secondary antibody for 10 min at room temperature. Opal fluorophores were pipetted onto each slide for 10 min at room temperature, and the slides were microwaved to strip the primary and secondary antibodies (Step 1). Then, we repeated the same protocol using the next primary antibody targets (Steps 2-6). Finally, DAPI was pipetted onto each slide for 10 minutes at room temperature (Step 7). The slides were

covered with BDHM-BD-FM-1, and images were taken using a Vectra Polaris automated quantitative pathology system. The images were analyzed by inForm 2.8.0 software (PerkinElmer, Waltham, USA).

Macrophage and neutrophil isolation

Mouse bone marrow was flushed from the femurs and washed through a 70 μm cell filter and red blood cells were lysed in lysis buffer. The resulting cells were cultured in RPMI 1640 medium (Gibco) supplemented with 10% fetal bovine serum (Gibco), 20 ng/ml macrophage colony-stimulating factor (Peprotech), and 2% penicillin/streptomycin (Gibco), and grown in 5% CO_2 at 37 °C. Then, half of the medium was changed every 1d until the 7d. At 8d, added 1, 2μM MCC950 (MCE, HY-12815A) to the cell plate, 2 hours later, infecting macrophage with 0.0001 MOI P7, the level of Lcn2 protein expression was quantified in the supernatant collected after 24 hours.

Macrophages from the lung were sorted by EasySep™ Mouse F4/80 Positive Selection Kit (STEMCELL,#100-0659), and neutrophils from bone marrow were sorted by EasySep™ Mouse Neutrophil Enrichment Kit (STEMCELL, #19762), according to the manufacturer's instructions.

Neutrophil adhesion

The neutrophil sorting from bone marrow was incubated with RatioWorks™ BCFL, AM *Superior replacement for BCECF*(AAT Bioquest, #21190) in 5% CO_2 at 37 °C. After 30 minutes, BCECF/AM fluorescently labeled PMNs were washed twice with PBS to remove the unbound dye, and the cells were re-suspended in a DMEM culture medium containing 10%FBS. 2×10^5 neutrophil were added to the PMVEC, and 1mg/mL Lcn2 (RD, 1857-LC-50) was added at the same time, after 2 hours, the culture medium was carefully sucked out, after washing with PBS, the non-adherent PMNs were removed. The cells were fixed with 4% paraformaldehyde at room temperature for 30 minutes. Images were finally captured using fluorescence microscopy(Leica).

In Vitro Vascular Permeability Assay

A total of 2.5×10^5 PMVECs was seeded into transwell inserts with 24 mm diameter, 0.4 μm pore size (Millipore, ECM644), and was allowed to grow in DMEM medium supplemented with 10% FBS to confluence. Upon the PMVEC monolayer growing to confluence, the PMNs were inoculated, together with 1mg/ml Lcn2. After 24 hours, transfer each insert to a fresh plate well in a new receiver tray, prepare a working solution of FITC-Dextran in media, and prepare a

sufficient volume of solution to add 150µl per insert. Protect the FITC-Dextran solution from light at all times. Add 500µl of media to each well of the receiver tray for each insert being analyzed. Incubate the plate, protected from light, for 20 minutes at room temperature. After 20 minutes, remove 100µl of the media from each well of the receiver tray, and transfer to wells of the black 96-well opaque plate provided for fluorescence measurement. Read the plate using a fluorescence plate reader(BMG LABTACH PHERAstar FSX) with filters appropriate for 485 nm and 535nm excitation and emission, respectively.

Statistical analysis

All data were analyzed with GraphPad Prism 8.0 software. Statistical significance was determined by performing an unpaired two-tailed Student’s t-test to compare two groups or by conducting one-way ANOVA when comparing three groups. The level of statistical significance was determined as $^*p < 0.05$, $^{**}p < 0.01$, $^{***}p < 0.001$.

References

Love, M.I., Huber, W., and Anders, S. (2014). Moderated estimation of fold change and dispersion for RNA-seq data with DESeq2. Genome Biol 15, 550.

Yu, G., and He, Q.Y. (2016). ReactomePA: an R/Bioconductor package for reactome pathway analysis and visualization. Mol Biosyst 12, 477-479.

Yu, G., Wang, L.G., Han, Y., and He, Q.Y. (2012). clusterProfiler: an R package for comparing biological themes among gene clusters. Omics 16, 284-287.

Supplemental Figure Legends

Fig. S1 Symptoms following infection with P0 strain and P7 strain at $1{\times}10^{3.9}$ $TCID_{50}$ in mice. (A**)** Diagram illustrating the continuous passage of mice, denoted as P0 in the primary generation and Pn in the nth generation. (B) Mutation site of S segment of P7 strain. (C) Experimental design and sample collection. After infecting BALB/c mice with P0 strain and P7 strain, we collected lungs at 3, 5, and 7 days for viral load quantification, HE staining, and detection of inflammatory cytokine levels. (D)Weight change rate within 14 dpi in infected mice (n=5). At 3, 5, 7dpi. (E) Viral RNA loads from the lung were detected by qRT-PCR in infected mice (n=3). (F) Histopathological changes in the lungs were observed by hematoxylin and eosin (H&E) staining in infected mice. Red arrows indicate hyaline membrane. (G) Pathological score of the lung in infected mice (n=3). Black bar=100 μm. Significant differences are indicated with asterisks(*$p < 0.05$;**$p < 0.01$; ***$p < 0.001$; Student's t-test).

Fig. S2 Expression levels of inflammatory cytokines and chemokines in lung after infection. (A) Inflammatory cytokines and chemokines mRNA expression in the lungs of mice infected with P0 strain and P7 strain were detected by qRT-PCR at 3, 5, and 7dpi (n=3). (B) Inflammatory cytokines and chemokines protein expression in the lungs of mice infected with P0 strain and P7 strain were detected by the LEGENDplex™ Mouse Inflammation Panel at 3, 5, 7dpi (n=3). (C) Inflammatory cytokines and chemokines protein expression in the serum of mice infected with P0 strain and P7 strain were detected by the LEGENDplex™ Mouse Inflammation Panel at 3, 5, 7dpi (n=3). Significant differences are indicated with asterisks(*$p < 0.05$; **$p <$ 0.01; Student's t-test).

Fig. S3 mRNA transcriptome results of 3, 5, and 7dpi in lung of mice infected with $1 \times 10^{3.9}$ $TCID_{50}$ of P0 strain and P7 strain. (A) PCA plot on differentially expressed genes. (B) Volcano plot displaying the statistical significance (p-value) versus magnitude of change (Fold change) of genes in P7 compared with P0. The horizontal dashed line represents $-\log_{10}(0.05)$, and the vertical dashed line represents $\pm\log_2(1.5)$. (C) For all pathways related to the IL-1β gene, the red section on the left represents the $-\log_{10}(p\text{-value})$ of the pathway, and the blue section on the right represents the mean $-\log_{10}(p\text{-value})$ of all significantly differentially expressed genes in the pathway. (D) GO enrichment analysis for genes that were differentially expressed in P7 versus

P0 at 3, 5, 7 dpi. (E) KEGG enrichment analysis results of differentially expressed genes between P7 and P0 at 3, 5, 7 dpi.

Fig. S4 mRNA expression of NLRP3 signaling pathway in lung macrophages and inflammatory cytokines and adhesion factors in Lcn2 stimulated endothelial cells. (A) The NLRP3, ASC, Caspase1, IL-1β mRNA expression levels in sorted lung macrophages from each group of mice (Control, P0, and P7 group) were detected through qRT-PCR (*n*=3). (B) Lcn2 stimulates PMVEC to secrete mRNA levels of inflammatory cytokines (*n*=3). (C) Lcn2 stimulates PMVEC to secrete mRNA levels of ICAM1, VCAM1 and CD62E (*n*=3). Significant differences are indicated with asterisks(*$p < 0.05$; **$p < 0.01$; Student's t-test or one-way ANOVA analysis).

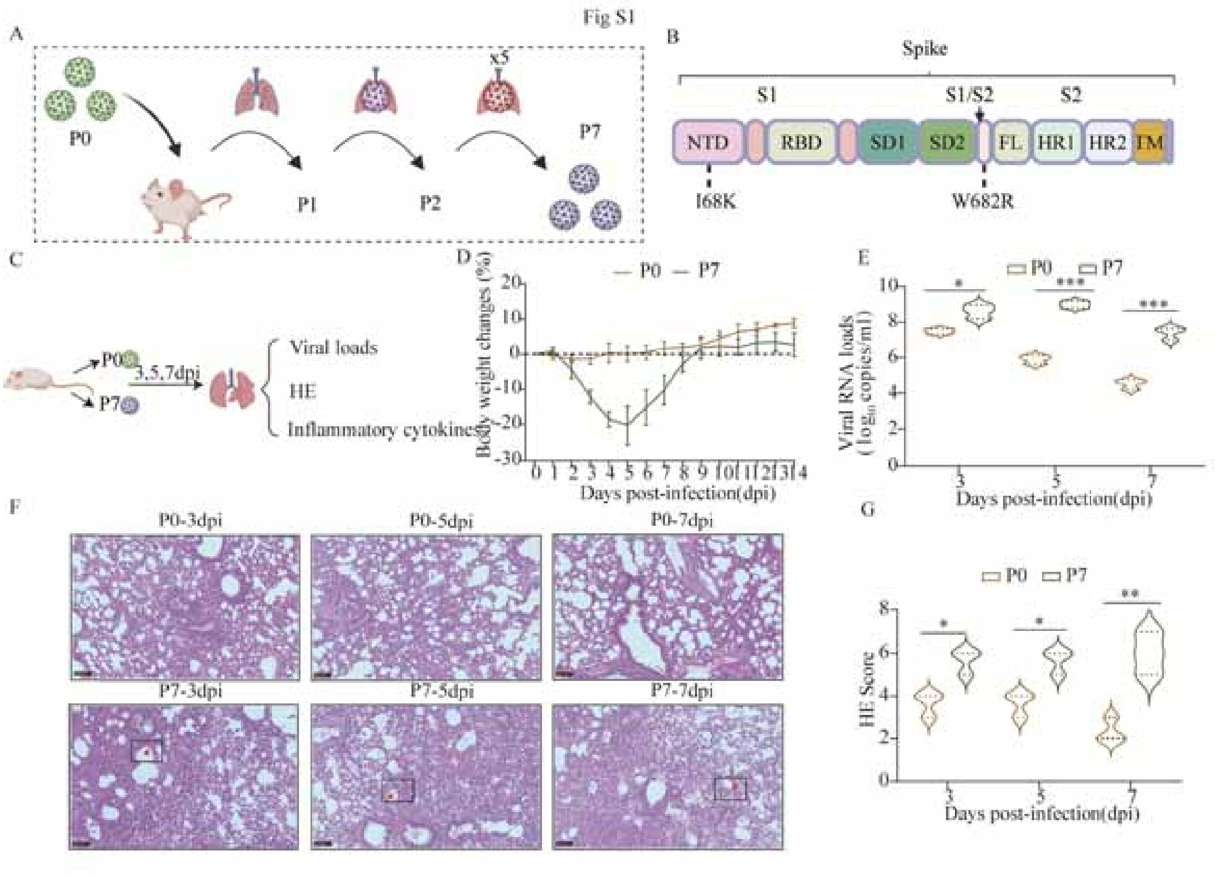
Fig S1
A
P0
x5
P7
P1
P2
B
Spike
S1
S1/S2
S2
NTD
RBD
SD1
SD2
FL
HR1
HR2
TM
I68K
W682R
C
P0
3,5,7dpi
P7
Viral loads
HE
Inflammatory cytokines
D
P0
P7
Body weight changes (%)
Days post-infection(dpi)
E
P0
P7
Viral RNA loads
(log10 copies/ml)
Days post-infection(dpi)
F
P0-3dpi
P0-5dpi
P0-7dpi
P7-3dpi
P7-5dpi
P7-7dpi
G
P0
P7
HE Score
Days post-infection(dpi)

Fig S2

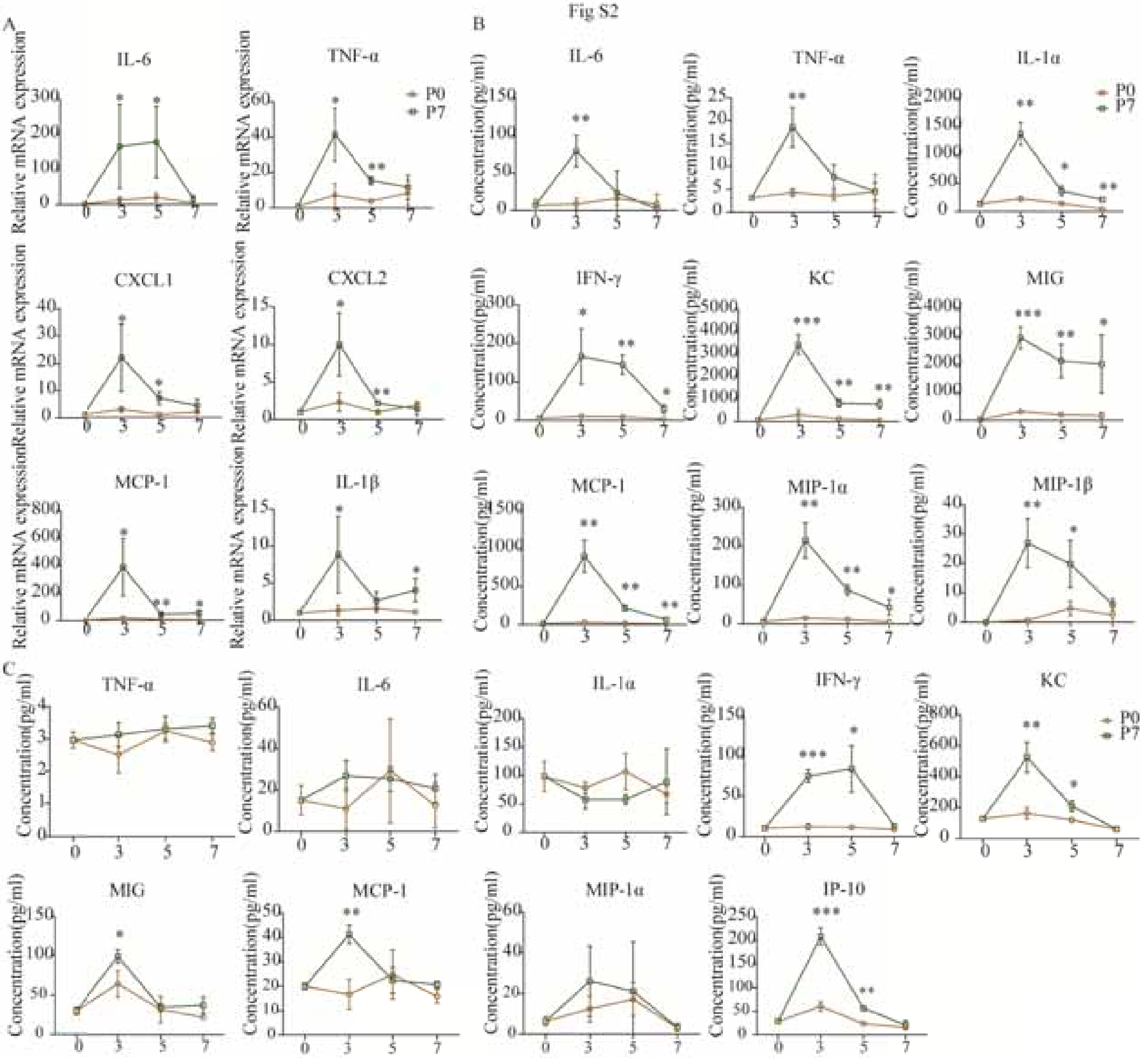
A
B
C
IL-6
TNF-α
IL-1α
CXCL1
CXCL2
IFN-γ
KC
MIG
MCP-1
IL-1β
MIP-1α
MIP-1β
IP-10
P0
P7
Relative mRNA expression
Concentration(pg/ml)

Fig S3

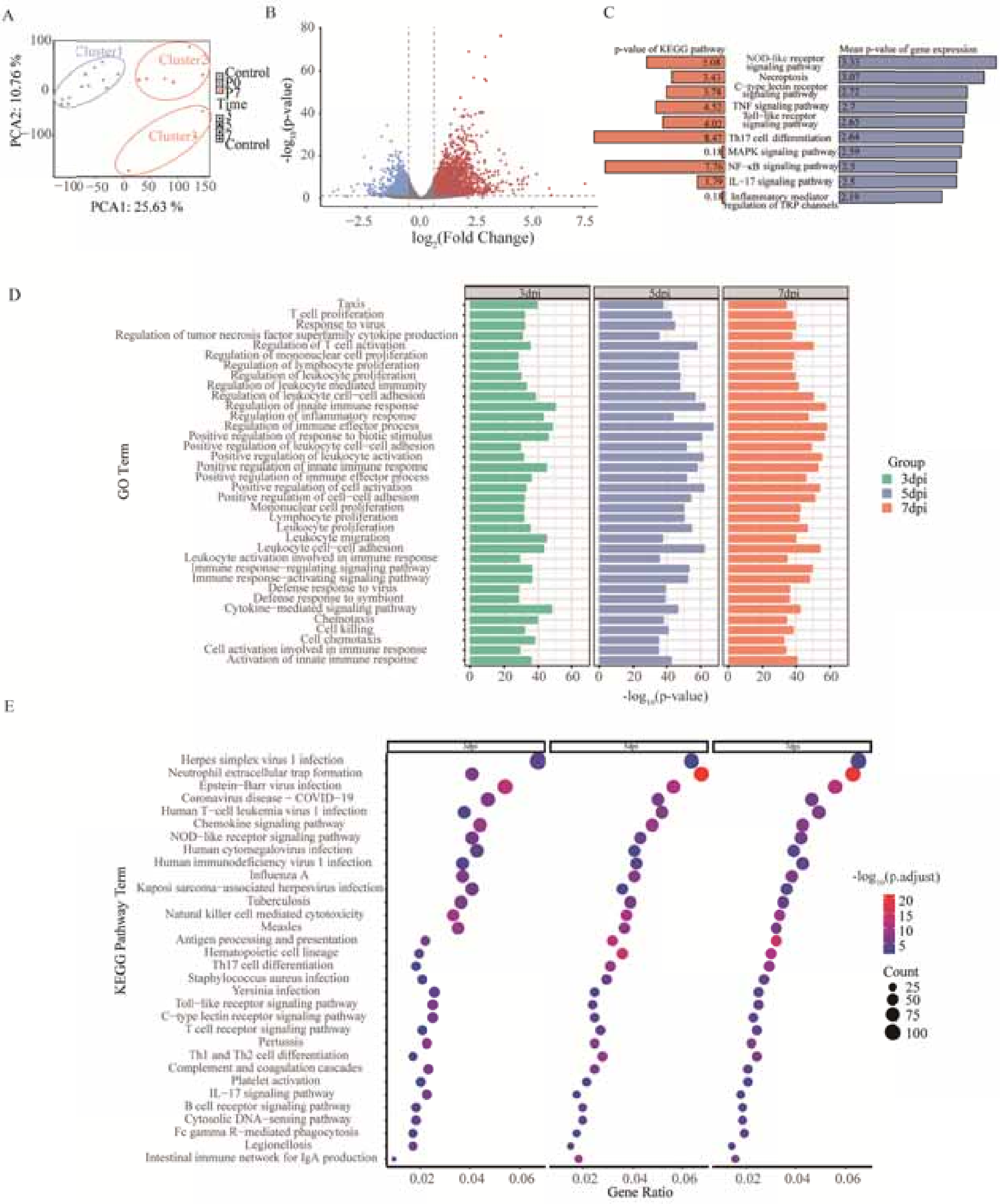
A
Cluster1
Cluster2
Cluster3
PCA1: 25.63 %
PCA2: 10.76 %
Control
P0
P7
Time
Control
B
-log10(p-value)
log2(Fold Change)
C
p-value of KEGG pathway
Mean p-value of gene expression
NOD-like receptor signaling pathway
Necroptosis
C-type lectin receptor signaling pathway
TNF signaling pathway
Toll-like receptor signaling pathway
Th17 cell differentiation
0.18
MAPK signaling pathway
NF-κB signaling pathway
IL-17 signaling pathway
0.18
Inflammatory mediator regulation of TRP channels
D
3dpi
5dpi
7dpi
Taxis
T cell proliferation
Response to virus
Regulation of tumor necrosis factor superfamily cytokine production
Regulation of T cell activation
Regulation of mononuclear cell proliferation
Regulation of lymphocyte proliferation
Regulation of leukocyte proliferation
Regulation of leukocyte mediated immunity
Regulation of leukocyte cell-cell adhesion
Regulation of innate immune response
Regulation of inflammatory response
Regulation of immune effector process
Positive regulation of response to biotic stimulus
Positive regulation of leukocyte cell-cell adhesion
Positive regulation of leukocyte activation
Positive regulation of innate immune response
Positive regulation of immune effector process
Positive regulation of cell activation
Positive regulation of cell-cell adhesion
Mononuclear cell proliferation
Lymphocyte proliferation
Leukocyte proliferation
Leukocyte migration
Leukocyte cell-cell adhesion
Leukocyte activation involved in immune response
Immune response-regulating signaling pathway
Immune response-activating signaling pathway
Defense response to virus
Defense response to symbiont
Cytokine-mediated signaling pathway
Chemotaxis
Cell killing
Cell chemotaxis
Cell activation involved in immune response
Activation of innate immune response
GO Term
-log10(p-value)
Group
3dpi
5dpi
7dpi
E
Herpes simplex virus 1 infection
Neutrophil extracellular trap formation
Epstein-Barr virus infection
Coronavirus disease - COVID-19
Human T-cell leukemia virus 1 infection
Chemokine signaling pathway
NOD-like receptor signaling pathway
Human cytomegalovirus infection
Human immunodeficiency virus 1 infection
Influenza A
Kaposi sarcoma-associated herpesvirus infection
Tuberculosis
Natural killer cell mediated cytotoxicity
Measles
Antigen processing and presentation
Hematopoietic cell lineage
Th17 cell differentiation
Staphylococcus aureus infection
Yersinia infection
Toll-like receptor signaling pathway
C-type lectin receptor signaling pathway
T cell receptor signaling pathway
Pertussis
Th1 and Th2 cell differentiation
Complement and coagulation cascades
Platelet activation
IL-17 signaling pathway
B cell receptor signaling pathway
Cytosolic DNA-sensing pathway
Fc gamma R-mediated phagocytosis
Legionellosis
Intestinal immune network for IgA production
KEGG Pathway Term
Gene Ratio
-log10(p.adjust)
Count

Fig S4

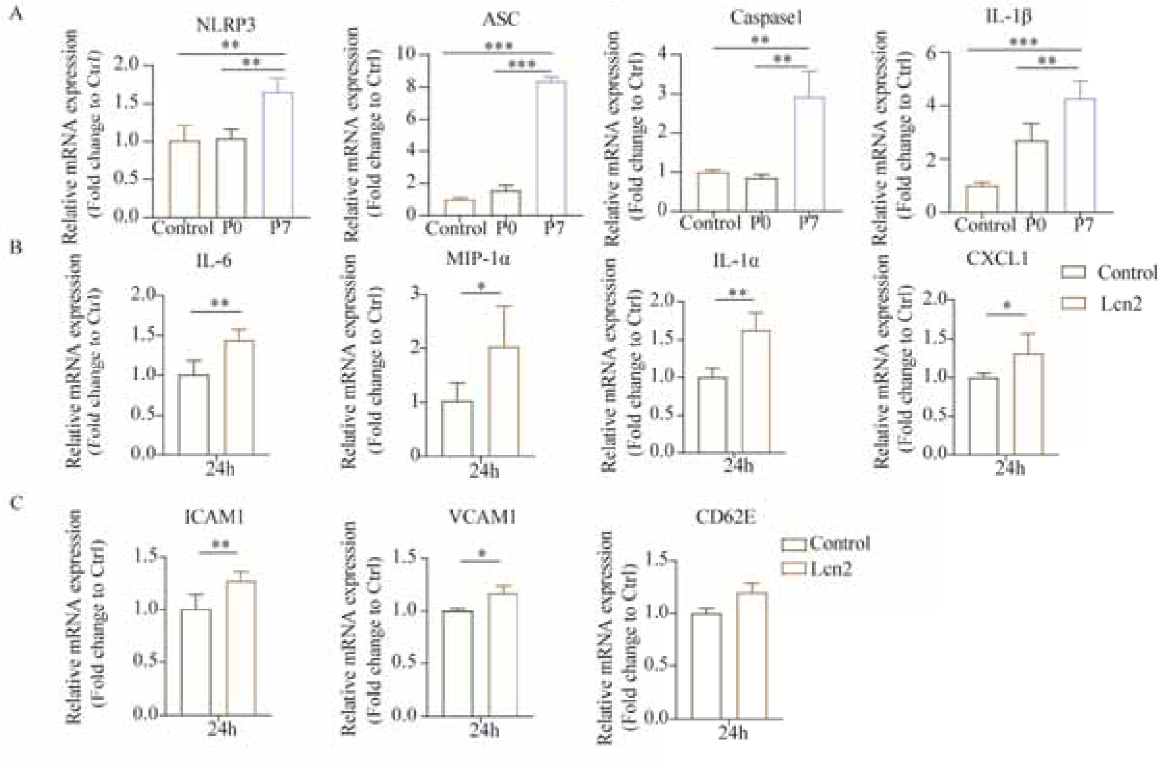
A
NLRP3
ASC
Caspase1
IL-1β
Relative mRNA expression (Fold change to Ctrl)
Control
P0
P7
B
IL-6
MIP-1α
IL-1α
CXCL1
Control
Lcn2
24h
C
ICAM1
VCAM1
CD62E

TABLE S1 Primer sequences used for qRT-PCR analysis		
Gene	Forward(5'-3')	Reverse(5'-3')
IL-6	CTGCAAGAGACTTCCATCCAG	AGTGGTATAGACAGGTCTGTTGG
TNF-α	CAGGCGGTGCCTATGTCTC	CGATCACCCCGAAGTTCAGTAG
CXCL1	ACTGCACCCAAACCGAAGTC	TGGGGACACCTTTTAGCATCTT
CXCL2	CCAACCACCAGGCTACAGG	GCGTCACACTCAAGCTCTG
MCP-1	AGTAGGCTGGAGAGCTACAA	GTATGTCTGGACCCATTCCTTC
IL-1β	CAACCAACAAGTGATATTCTCCATG	GATCCACACTCTCCAGCTGCA
Lcn2	TGGCCCTGAGTGTCATGTG	CTCTTGTAGCTCATAGATGGTGC
NLRP3	ATCAACAGGCGAGACCTCTG	GTCCTCCTGGCATACCATAGA
ASC	GACAGTGCAACTGCGAGAAG	CGACTCCAGATAGTAGCTGACAA
Caspase1	ACAAGGCACGGGACCTATG	TCCCAGTCAGTCCTGGAAATG
MIP-1α	TTCTCTGTACCATGACACTCTGC	CGTGGAATCTTCCGGCTGTAG
IL-1α	TCTCAGATTCACAACTGTTCGTG	AGAAAATGAGGTCGGTCTCACTA
ICAM1	GTGATGCTCAGGTATCCATCCA	CACAGTTCTCAAAGCACAGCG
VCAM1	TTGGGAGCCTCAACGGTACT	GCAATCGTTTTGTATTCAGGGGA
CD62E	ATGAAGCCAGTGCATACTGTC	CGGTGAATGTTTCAGATTGGAGT
β-actin	CAACGAGCGGTTCCGATG	GCCACAGGATTCCATACCCA
SARS-CoV-2	TCGTTTCGGAAGAGACAGGT	GCGCAGTAAGGATGGCTAGT

Table S1.

Primer sequences used for qRT-PCR analysis.

Fig S1

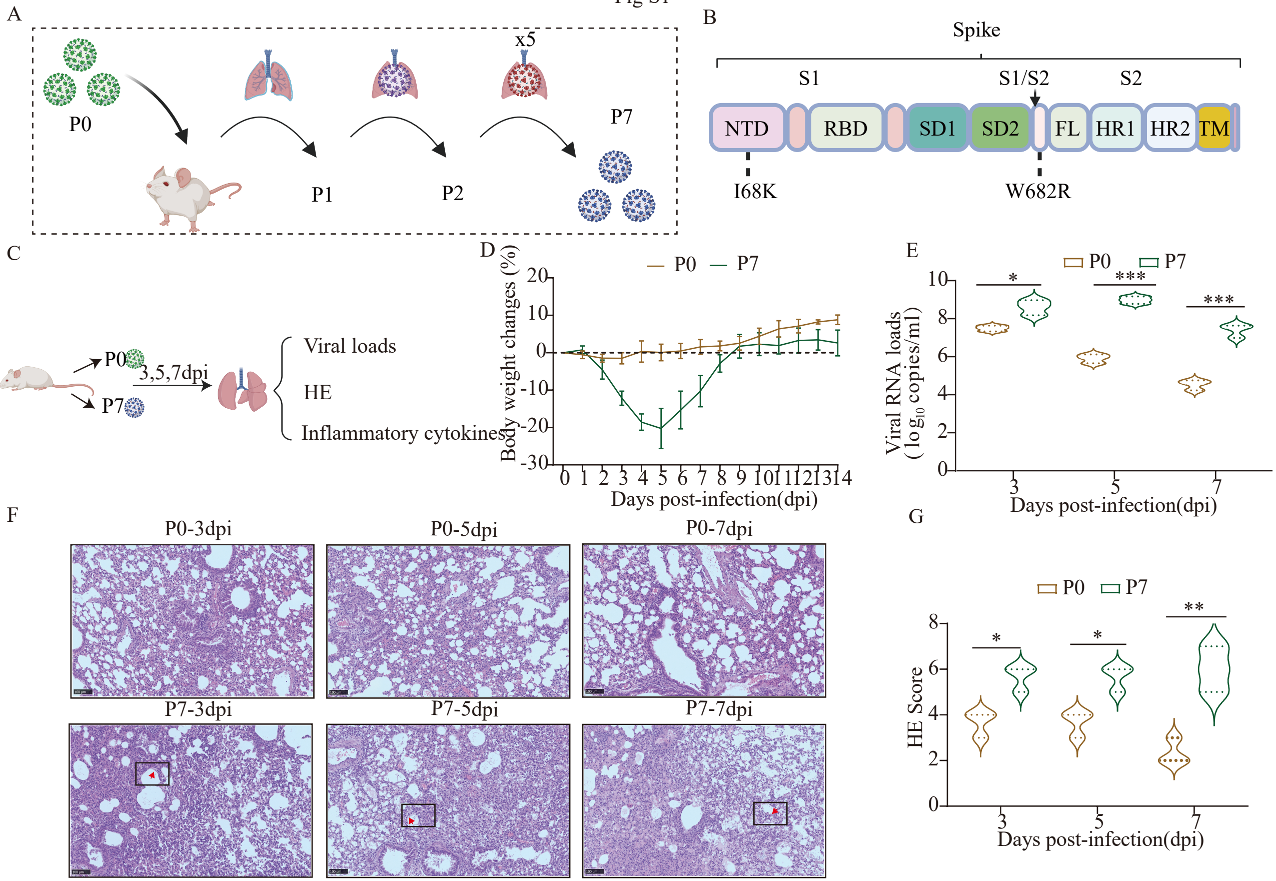
A
P0
P1
P2
x5
P7
B
Spike
S1
S1/S2
S2
NTD
RBD
SD1
SD2
FL
HR1
HR2
TM
I68K
W682R
C
P0
P7
3,5,7dpi
Viral loads
HE
Inflammatory cytokines
D
P0
P7
Body weight changes (%)
Days post-infection(dpi)
E
P0
P7
Viral RNA loads (log10 copies/ml)
Days post-infection(dpi)
F
P0-3dpi
P0-5dpi
P0-7dpi
P7-3dpi
P7-5dpi
P7-7dpi
G
P0
P7
HE Score
Days post-infection(dpi)

Fig S2

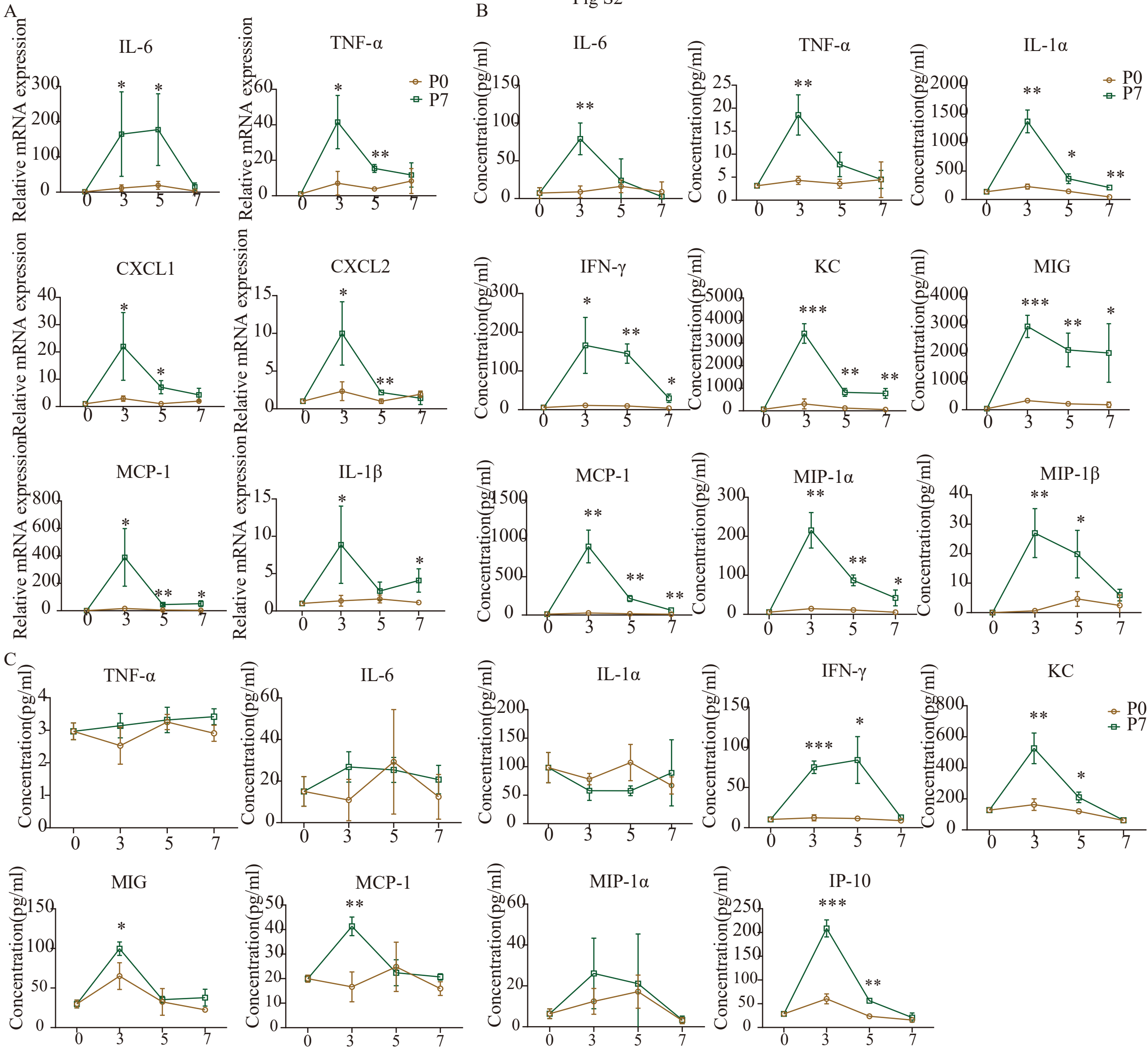
A
IL-6
TNF-α
CXCL1
CXCL2
MCP-1
IL-1β
Relative mRNA expression
P0
P7
B
IL-6
TNF-α
IL-1α
IFN-γ
KC
MIG
MCP-1
MIP-1α
MIP-1β
Concentration(pg/ml)
C
TNF-α
IL-6
IL-1α
IFN-γ
KC
MIG
MCP-1
MIP-1α
IP-10

Fig S3

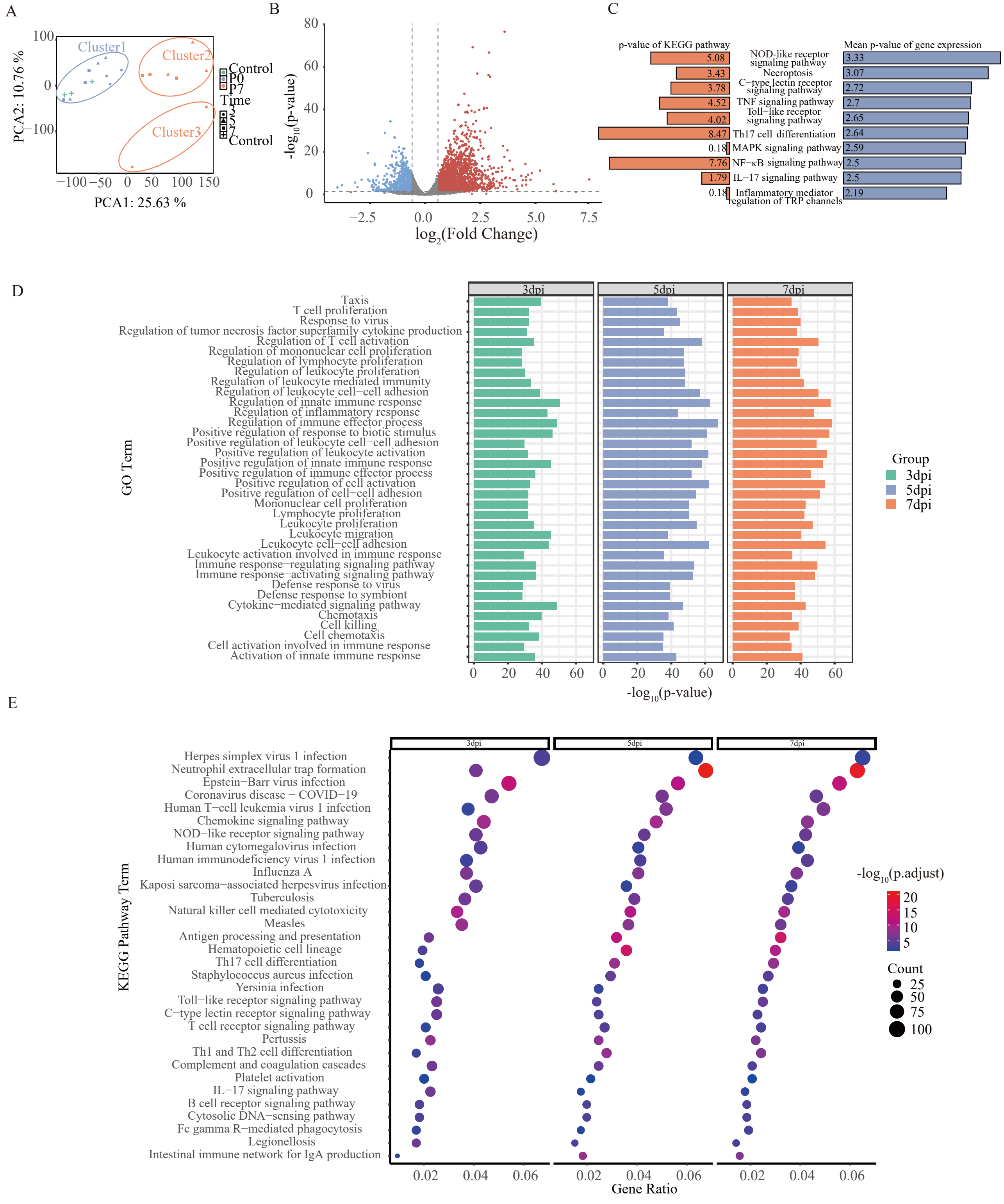

A
Cluster1
Cluster2
Cluster3
Control
P0
P7
Time
3
5
7
Control
PCA1: 25.63 %
PCA2: 10.76 %
B
$-log_{10}$(p-value)
log_2(Fold Change)
C
p-value of KEGG pathway
Mean p-value of gene expression
NOD-like receptor signaling pathway 5.08 3.33
Necroptosis 3.43 3.07
C−type lectin receptor signaling pathway 3.78 2.72
TNF signaling pathway 4.52 2.7
Toll−like receptor signaling pathway 4.02 2.65
Th17 cell differentiation 8.47 2.64
MAPK signaling pathway 0.18 2.59
NF−κB signaling pathway 7.76 2.5
IL−17 signaling pathway 1.79 2.5
Inflammatory mediator regulation of TRP channels 0.18 2.19
D
3dpi
5dpi
7dpi
GO Term
Taxis
T cell proliferation
Response to virus
Regulation of tumor necrosis factor superfamily cytokine production
Regulation of T cell activation
Regulation of mononuclear cell proliferation
Regulation of lymphocyte proliferation
Regulation of leukocyte proliferation
Regulation of leukocyte mediated immunity
Regulation of leukocyte cell−cell adhesion
Regulation of innate immune response
Regulation of inflammatory response
Regulation of immune effector process
Positive regulation of response to biotic stimulus
Positive regulation of leukocyte cell−cell adhesion
Positive regulation of leukocyte activation
Positive regulation of innate immune response
Positive regulation of immune effector process
Positive regulation of cell activation
Positive regulation of cell−cell adhesion
Mononuclear cell proliferation
Lymphocyte proliferation
Leukocyte proliferation
Leukocyte migration
Leukocyte cell−cell adhesion
Leukocyte activation involved in immune response
Immune response−regulating signaling pathway
Immune response−activating signaling pathway
Defense response to virus
Defense response to symbiont
Cytokine−mediated signaling pathway
Chemotaxis
Cell killing
Cell chemotaxis
Cell activation involved in immune response
Activation of innate immune response
$-log_{10}$(p-value)
Group
3dpi
5dpi
7dpi
E
KEGG Pathway Term
Herpes simplex virus 1 infection
Neutrophil extracellular trap formation
Epstein−Barr virus infection
Coronavirus disease − COVID−19
Human T−cell leukemia virus 1 infection
Chemokine signaling pathway
NOD−like receptor signaling pathway
Human cytomegalovirus infection
Human immunodeficiency virus 1 infection
Influenza A
Kaposi sarcoma−associated herpesvirus infection
Tuberculosis
Natural killer cell mediated cytotoxicity
Measles
Antigen processing and presentation
Hematopoietic cell lineage
Th17 cell differentiation
Staphylococcus aureus infection
Yersinia infection
Toll−like receptor signaling pathway
C−type lectin receptor signaling pathway
T cell receptor signaling pathway
Pertussis
Th1 and Th2 cell differentiation
Complement and coagulation cascades
Platelet activation
IL−17 signaling pathway
B cell receptor signaling pathway
Cytosolic DNA−sensing pathway
Fc gamma R−mediated phagocytosis
Legionellosis
Intestinal immune network for IgA production
Gene Ratio
$-log_{10}$(p.adjust)
Count
25
50
75
100

Fig S4

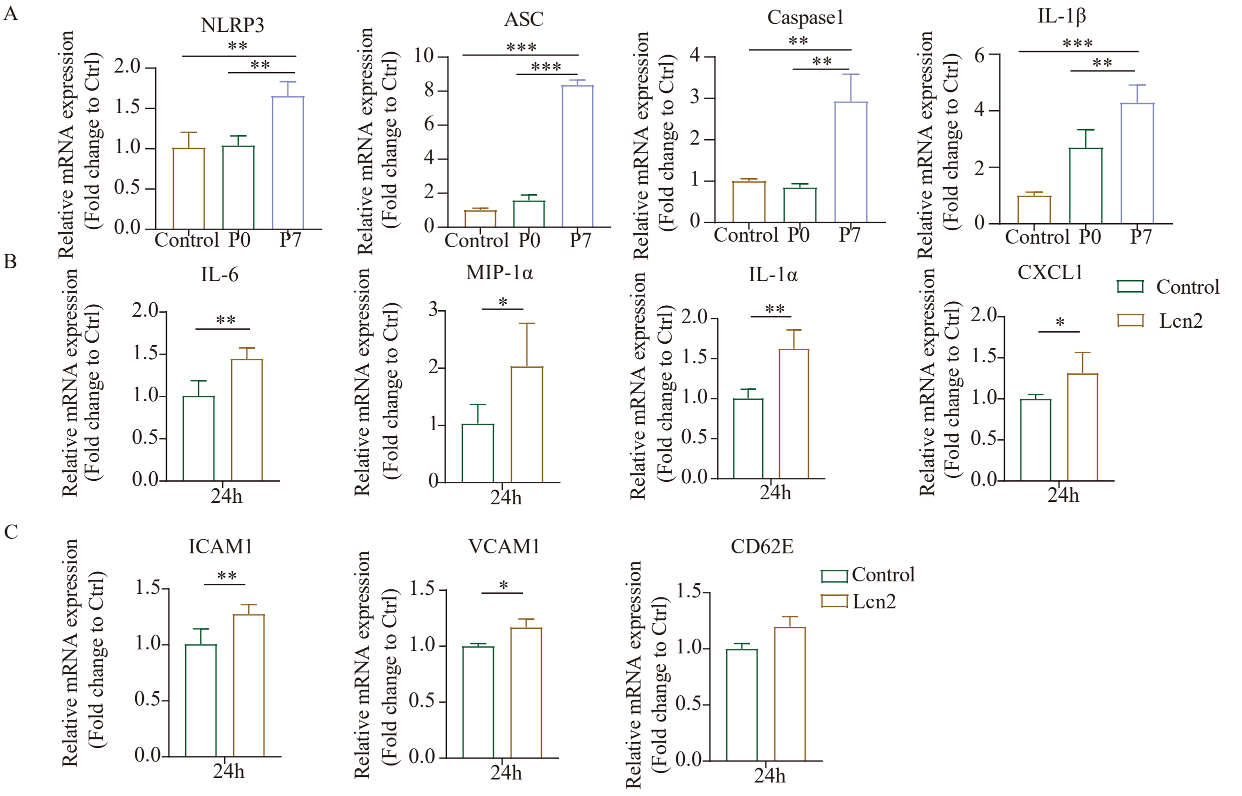
A
NLRP3
ASC
Caspase1
IL-1β
Relative mRNA expression
(Fold change to Ctrl)
Control
P0
P7
B
IL-6
MIP-1α
IL-1α
CXCL1
24h
Control
Lcn2
C
ICAM1
VCAM1
CD62E
24h
Control
Lcn2
